# In Vitro Generation of BK polyomavirus-specific T cells for adoptive cell therapy in refractory cystitis hemorrhagic patients after hematopoietic stem cell transplantation

**DOI:** 10.1186/s12865-022-00497-1

**Published:** 2022-06-10

**Authors:** Maryam Mohammadi Najafabadi, Masoud Soleimani, Mohammad Ahmadvand, Mina Soufi Zomorrod, Seied Asadollah Mousavi

**Affiliations:** 1 Department of Hematology, Faculty of Medical Science, Tarbiat Modares University, Tehran, Iran; 2 Research Institute for Oncology, Hematology and Cell Therapy, Shariati Hospital, Tehran University of Medical Sciences, Tehran, Iran; 3 Applied Cell Sciences Department, Faculty of Medical Sciences, Tarbiat Modares University, Tehran, Iran

**Keywords:** BK virus, Hematopoietic stem cell transplantation, Cystitis hemorrhagic, Virus specific T cell, Adoptive cell therapy

## Abstract

**Introduction:**

BKPyV associated hemorrhagic cystitis (BKPyV-HC) is a major and prevalent outcome of hematopoietic stem cell transplantation (HSCT) with no standard treatment option. Adoptive T cell therapy (ACT) against transplant-associated viruses has shown promising potential. We sought to produce virus-specific T cells (VSTs) against BKPyV with the aim of treating refractory HSCT-associated HC.

**Methods:**

Peripheral blood mononuclear cells (PBMC) from healthy donors were isolated by Ficoll-Hypaque density gradient centrifugation. BKPyV-pulsed, monocyte-derived dendritic cells (mo-DCs) and T cells were co-cultured and expanded over 2–3 weeks with the addition of IL-2. The T cells were examined for various functional assays.

**Results:**

Comparison analysis of Carboxyfluorescein diacetate succinimidyl ester (CFSE) indicated that the percentage of proliferated cells were significantly higher in donors (49.62 ± 7.09%) than controls (7.96 ± 4.55%). Furthermore, expanded T cells exhibited specificity to BKPyV antigens by IFN-γ ELISPOT assay. The expanded cells showed cytotoxic function versus human lymphoblastoid cell line (LCL). Final VST products mainly comprised of CD8/CD69 double-positive T cells, which were significantly higher in donors (46.8 ± 7.1%) than controls (16.91 ± 3.40%).

**Conclusion:**

In this study we demonstrated the feasibility of producing functional BKPyV-specific T cells in healthy donors using BKPyV PepMixes. These functional cells were able to proliferate and produce IFN-γ cytokine in response to BKPyV PepMixes. In addition, these T cells had cytotoxic ability against BKPyV antigen-expressing target cells.

## Introduction

Hematopoietic stem cell transplantation (HSCT) is considered a therapeutic option for a large group of malignant and nonmalignant disorders. However, the transplantation results can be undermined by a variety of transplant-associated complications [1–4]. Infections that can be caused by various types of microorganisms are a main cause for mortality and morbidity during the immunodeficiency phase of HSCT. Overall, 11% of post-HSCT deaths are triggered by infections, with one-third of these being viral infections [5]. One such challenging virus is the human polyomavirus BK (BKPyV), which can cause hemorrhagic cystitis (HC) [3, 5]. HC symptoms include hematuria, dysuria, burning during urination, increased urinary frequency, abdominal or suprapubic pain, urinary obstruction, and renal or bladder damage [6–8]. HC is classified into two groups according to the time of incidence: early-onset HC, occurs during the first week after HSCT and late-onset HC, which arises between 2 to 8 weeks after HSCT. The latter is associated essentially with certain viruses such as adenovirus type I and II, cytomegalovirus (CMV), and BK and JC polyoma virus [8, 9]. It has been shown that BKPyV viruria occurs in 25–100% of stem cell transplanted patients, which can lead to BKPyV associated HC (BKPyV-HC) in up to 40% of the patients [10]. Still, there is no approved and standard treatment practice for BKPyV-HC. Supportive care that are currently used mostly include surgical procedures and pharmacological treatments. Most cases of BKPyV-HC are mild, self-limited and treatable, but refractory patients or severe episodes may require further care [11].

The typical clinical method upon documentation of BKPyV viremia after HSCT is gradual reduction of immunosuppression regimen which may increase the risk of acute rejection and exacerbating graft-versus-host disease (GVHD) [12]. Among all pharmacological therapies used to treat BKPyV-HC, Cidofovir (CDV) has the highest specificity against BKV. It is presently the frontline drug for the treatment of BKPyV-HC, but its intravenous application is often limited by nephrotoxicity especially in higher doses. This drug is not available in the majority of countries and it is an expensive drug [13–15]. Hence, it is believed that adoptive cell therapy, particularly virus-specific T cells (VSTs), might be a logical alternative to conventional treatments for patients with refractory HC [16–20]. Several methods have been employed to manufacture VSTs against various viruses which have been associated with remarkable successes. Overall 62%, 74% and 85% of patients responded to EBV-, Adv-, and CMV-specific T cell therapy, respectively [21]. Since VST infusion has been associated with notable improvement in post-transplant complications of these viruses, it can be readily applicable to many other viruses including BKPyV. VST infusion in the setting of BKPyV-HC is a novel therapeutic approach that is currently in its early clinical research.

As BKPyV is one of the basic causes of HC after HSCT and no standard and approved treatment protocol is available, our aim is the generation of VSTs against BKPyV with the hope that in the future it could be used to treat refractory HC patients after HSCT.

## Material and methods

### Isolation of peripheral blood mononuclear cells

Peripheral blood mononuclear cells (PBMCs) of healthy donors (*n* = 3) were isolated by Ficoll-Hypaque density gradient centrifugation after obtaining written, informed consent. The HLA typing of donors are presented in Table 1. HLA typing was performed by the Hematology-Oncology and Stem Cell Research center and was low resolution for class I loci and resolved to 4 digits at the DRB1 locus. The remaining cells were cryopreserved for further use. This study was reviewed and approved by the Ethics Committee of Tarbiat Modares University. All experiments were performed in accordance with the relevant guidelines and regulations.

**Table 1:** HLA typing of donors

	HLA-A	HLA-B	HLA-DRB1
Donor 1	01:01	08:51	0301
Donor 2	02:01	07:13	0901
Donor 3	02:01	08:49	0301

### Dendritic cell (DC) generation, maturation, and pulsing

DC were generated from peripheral blood monocytes. Briefly, PBMCs were washed three times with PBS (Sigma Aldrich, USA) and resuspended at the concentration of 3 $\times$ 10^6^ cell/mL in RPMI-1640 medium (Sigma Aldrich, USA) plus 1% FBS (Gibco, UK) and 2 mM L-glutamate (Gibco, UK) at 37 °C in a humidified CO2 incubator. After 2 h, non-adherent cells were removed by rinsing with PBS and human recombinant interleukin-4 (IL-4) (R&D Systems) at a final concentration of 1000 U/mL and human recombinant granulocyte-monocyte colony-stimulating factor (GM-CSF) at a final concentration of 800 U/mL (R&D Systems) were added After 5 days of incubation, monocyte-derived DCs (mo-DC) were matured with the addition of lipopolysaccharide (LPS) (Sigma Aldrich, USA) for 1 additional day. Mo-DCs were pulsed with BKPyV capsid protein VP1, and BKPyV-LargeT antigen PepMixes (JPT Peptide Technologies). PepMixes are overlapping 15-mer peptide mixtures that cover the entire length of both antigens.

### Stimulation and expansion of BKV-specific T cells

Irradiated (25 Gy) PepMix-pulsed mo-DCs were used to stimulate autologous T cells at a responder/stimulator ratio of 4:1. PepMix-pulsed mo-DCs and T cells were co-cultured in RPMI-1640 medium plus 10% FBS, and 2 mM L-glutamate and 1% pen/strep and expanded for 2–3 weeks with the addition of IL-2 (20 U/mL, R&D Systems) staring day 2, and IL-2 was replenished every 2 days. The responder cells were re-stimulated with PepMix-pulsed mo-DCs at a responder/stimulator ratio 4:1 on day 7 and 14. Co-culture of T cells with Actin-pulsed mo-DCs and T cells treated with IL-2 that had not received pulsed mo-DCs were used as controls. At the end of the co-culture, the generated lymphocytes were examined for functional assays.

### Carboxyfluorescein diacetate succinimidyl ester (CFSE) proliferation assay

CFSE assay was used to determine T cells' proliferation in response to BKPyV PepMixes. T cells were labeled with CFSE (BD Biosciences, US) according to the manufacturer's instructions and were co-cultured with PepMix-pulsed mo-DCs. Control cells that contained T cells treated with IL-2 and T cells treated with IL-2 with Actin-pulsed mo-DCs were also labeled with CFSE. After 7 days, cells were harvested and analyzed by flow cytometry. As controls, T cells at day 0, show the CFSE intensity of non-divided cells while the non-labeled cells show the auto-fluorescence of the cells and the limits of detectable cell divisions.

### IFN-γ ELISPOT

For ELISpot assays, multiscreen plates (Mabtech, Swedish) were coated with anti-human IFN-γ monoclonal antibody (Mabtech, Swedish) and stored at 4 °C overnight. After the blocking step, T cells were plated at 1 $\times$ 10^5^ cells/well and restimulated with BKPyV PepMixes at a concentration of 0.5 µg/ml. Each experiment was run in duplicate. Plates were incubated at 37 °C for 18–24 h. After incubation, plates were washed and incubated with the secondary biotin-conjugated anti-human IFN-γ monoclonal antibody (Mabtech, Swedish). After incubation with avidin: biotinylated horseradish peroxidase (HRP) complex, the AEC substrate (Mabtech, Swedish) was added to the plates. The plates were then dried overnight and quantified. The frequency of T cells specific for PepMixes was expressed as log_10_ IFN-γ ELISpots/10^6^ T lymphocytes.

### Cytotoxicity assay

Cytotoxicity was measured by Lactate dehydrogenase (LDH) Cytotoxicity Assay Kit (Sigma Aldrich, USA) according to the manufacturer's instructions. Briefly, autologous human lymphoblastoid cell line (LCL), was incubated with BKPyV PepMixes (0.5 nmol/peptide/mL) for 1–2 h. Target cells were incubated in 96-well plates with effector T cells in 20:1, 10:1, 5:1 and 1:1 effector/target cell ratios for 4 h at 37 °C. At the end of the incubation, the plate was centrifuged and 50µL of each sample medium was transferred to a 96-well plate and mixed with 50µL of Reaction Mixture. Plate was incubated at room temperature for half an hour. Finally, 50µL of Stop Solution was added to each sample well and absorbance was measured at 490 nm and 680 nm.

$$\% {\text{Cytotoxicity}} = \frac{{ {\text{Experimental value {-} Effector Cells Spontaneous Control}} {-} {\text{Target Cells Spontaneous Control}} }}{{{\text{Target Cell Maximum Control}} {-} {\text{Target Cells Spontaneous Control}}}} \times \, 100$$

### Flow cytometry

Staining of cell surface markers was performed with CD3-APC, CD4-Vioblue, CD8-Viogreen, CD56-PE and CD69-FITC (Miltenyi Biotec). Appropriate isotype-matched controls were used. Cells were examined with BD FACSLyric™ flow cytometer (Biosciences, US) and the data were analyzed with FlowJo 10.6.2.

### Data analysis

Results were evaluated using descriptive statistics (mean ± SD). Comparisons between responses to viral PepMixes and control were performed using a two sided unpaired t test. Each sample was analyzed in triplicate. *P* value < 0.05 was considered statistically significant. Analysis was done in GraphPad Prism 6.

## Results

### Successful T cell expansion in response to viral antigens

**Fig. 1:**
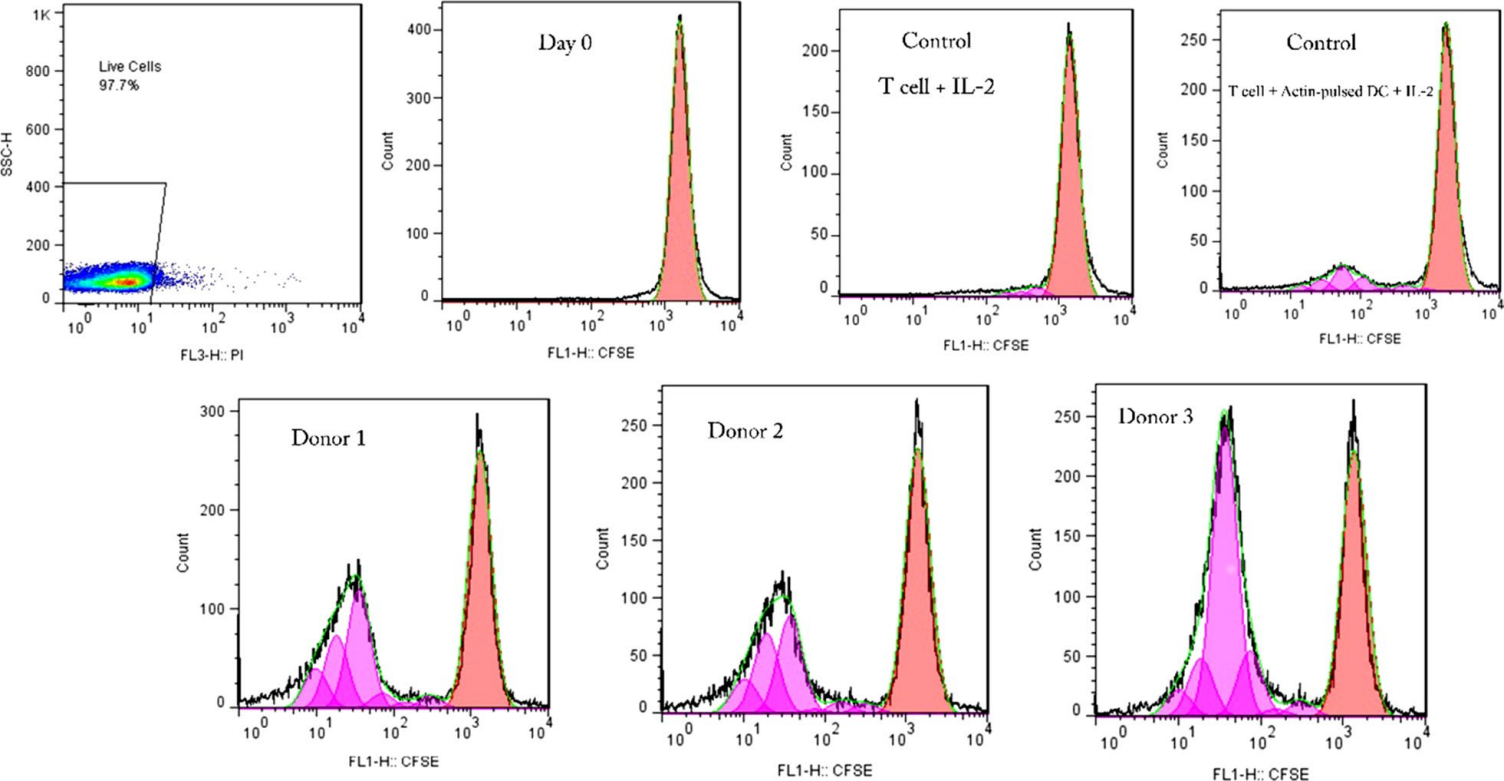
CFSE flow cytometric graphs of controls and donors (Only one graph is shown for each donor). T cells treated with IL-2 and T cells treated with IL-2 with Actin-pulsed mo-DCs are used as controls

**Fig. 2:**
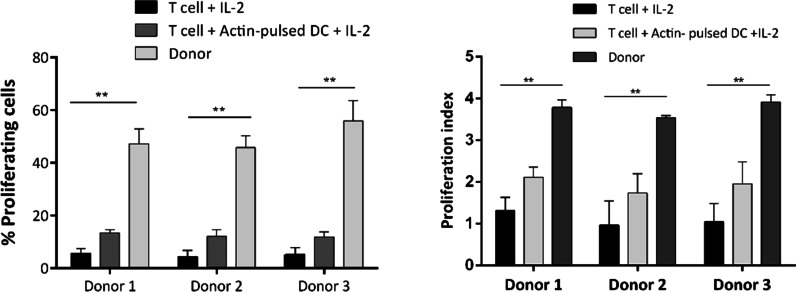
CFSE proliferation assay was used to determine the proliferation of T cells after co-culture with BKV PepMix-pulsed mo-DCs. This graph shows the percentage of proliferating cells and the proliferation index in each donor compared to both controls which are statistically significant (*p* value < 0.01). T cells treated with IL-2 and T cells co-cultured with Actin-pulsed mo-DCs are used as controls

We used the CFSE proliferation assay to investigate whether BKPyV PepMixes could stimulate lymphocyte proliferation (Fig. 1). The expansion of lymphocytes in each donor was examined against the control. The mean percentage of expanded cells was 47.17 ± 5.73%, 45.8 ± 4.45%, and 55.9 ± 7.64% for donor 1, 2, and 3, respectively. The mean expansion for T cells treated with IL-2 (control) and T cells co-cultured with Actin-pulsed mo-DCs (control), was 4.16 ± 2.11% and 11.76 ± 1.98%, respectively. The mean percentage of proliferated cells in donor samples was significantly increased compared to both controls (*p* value < 0.01). No significant difference was observed among donors. The proliferation index of donors and controls, defined as the fold expansion during culture, are depicted in Fig. 2.

### Increased IFN-γ-secreting VST numbers upon stimulation

**Fig. 3:**
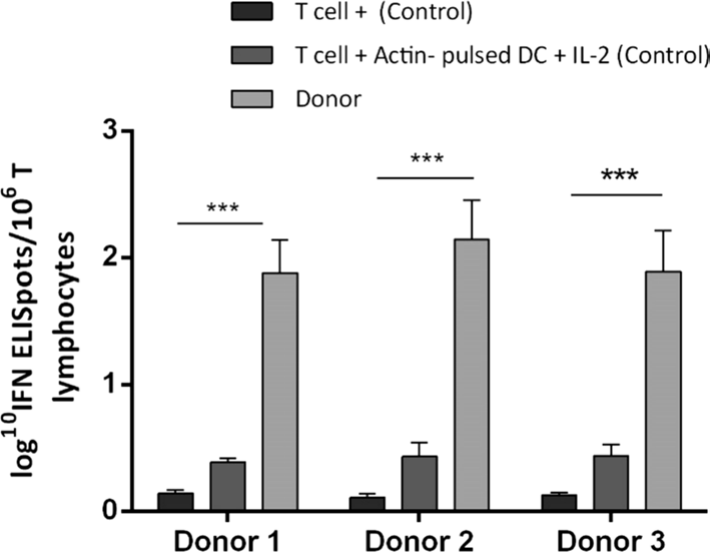
ELISPOT assay demonstrating the frequency and the specificity of generated T cells against BK viral PepMixes. The number of IFN-γ-producing lymphocytes in donors are significantly higher than both controls (*p* value < 0.001). T cells treated with IL-2 and T cells co-cultured with Actin-pulsed mo-DCs are used as controls

The ELISPOT technique was used to evaluate the frequency and the specificity of generated T cells against BKPyV PepMixes. The median of log_10_ IFN-γ ELISpots/10^6^ T cells in donors 1, 2, and 3, were 1.88 ± 0.26, 2.14 ± 0.31, and 1.89 ± 0.32, respectively. This was 0.12 ± 0.02 and 0.41 ± 0.08 for T cells treated with IL-2 (control) and T cells co-cultured with Actin-pulsed mo-DCs (control), respectively. Therefore, the number of antigen-specific T cells in the donors increased significantly compared to both controls (*p* value < 0.001). No significant difference was observed among donors (Fig. 3).

### Eradication of BKPyV-expressing target cells by generated T cells

**Fig. 4:**
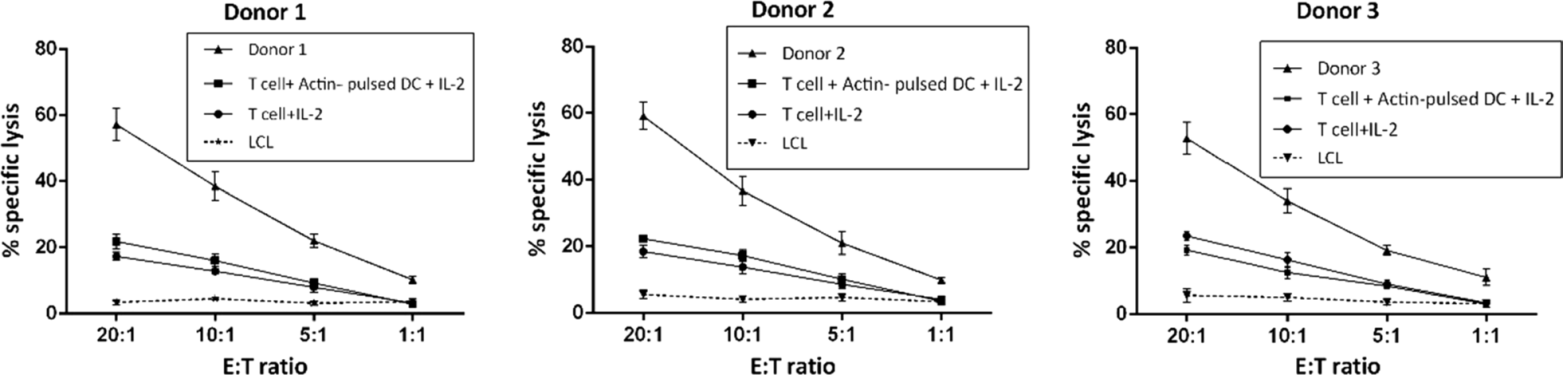
LDH Cytotoxicity Assay shows the percentage of target cell lysis for each donor in different E/T ratios. Human lymphoblastoid cell lines pulsed with BKV PepMixes were used as target cells. In all E/T ratios, the percentage of lysis in donors was statistically significant compared to both controls. T cells treated with IL-2 and T cells co-cultured with Actin-pulsed mo-DCs are used as controls

LDH cytotoxicity assay was performed to evaluate the ability of generated T cells for specific elimination of BKPyV antigen-expressing cells. We used human LCL pulsed with BKPyV PepMixes as target cells. We examined the percentage of target cell lysis for each donor in 20:1, 10:1, 5:1 and 1:1 E/T ratios. Each donor was examined in triplicate. The percentage of target cell lysis for donors and controls in each E/T ratios are presented in Table 2. The generated T cells in all three donors could kill target cells, which was significantly higher in donors than controls (*p* value < 0.05). At higher E/T ratios, the lysis rate of the target cells was higher in each donor. No significant difference was observed between donors. Figure 4 shows the results of cytotoxicity assays.

**Table 2:** The results of LDH cytotoxicity assay in each donor and controls against human lymphoblastoid cell line pulsed with BKPyV PepMixes

	T cell + BKPyV PepMix-pulsed mo-DC + IL-2			Controls	
E/T ratio	Donor 1 (%)	Donor 2 (%)	Donor 3 (%)	T cell + Actin-pulsed mo-DC + IL-2 (%)	T cell + IL-2 (%)
20:1	57.16 ± 4.83	59.16 ± 4.11	52.86 ± 4.71	23.56 ± 5.34	19.34 ± 5.10
10:1	38.53 ± 4.22	36.70 ± 4.33	34.06 ± 3.72	16.36 ± 3.48	12.56 ± 4.07
5:1	22.13 ± 1.95	21.01 ± 3.45	19.26 ± 1.40	9.10 ± 2.15	8.50 ± 2.47
1:1	10.33 ± 1.02	9.90 ± 0.80	11.10 ± 2.5	3.46 ± 1.13	3.07 ± 1.64

### Higher CD8/CD69 positive populations in the final product

**Fig. 5:**
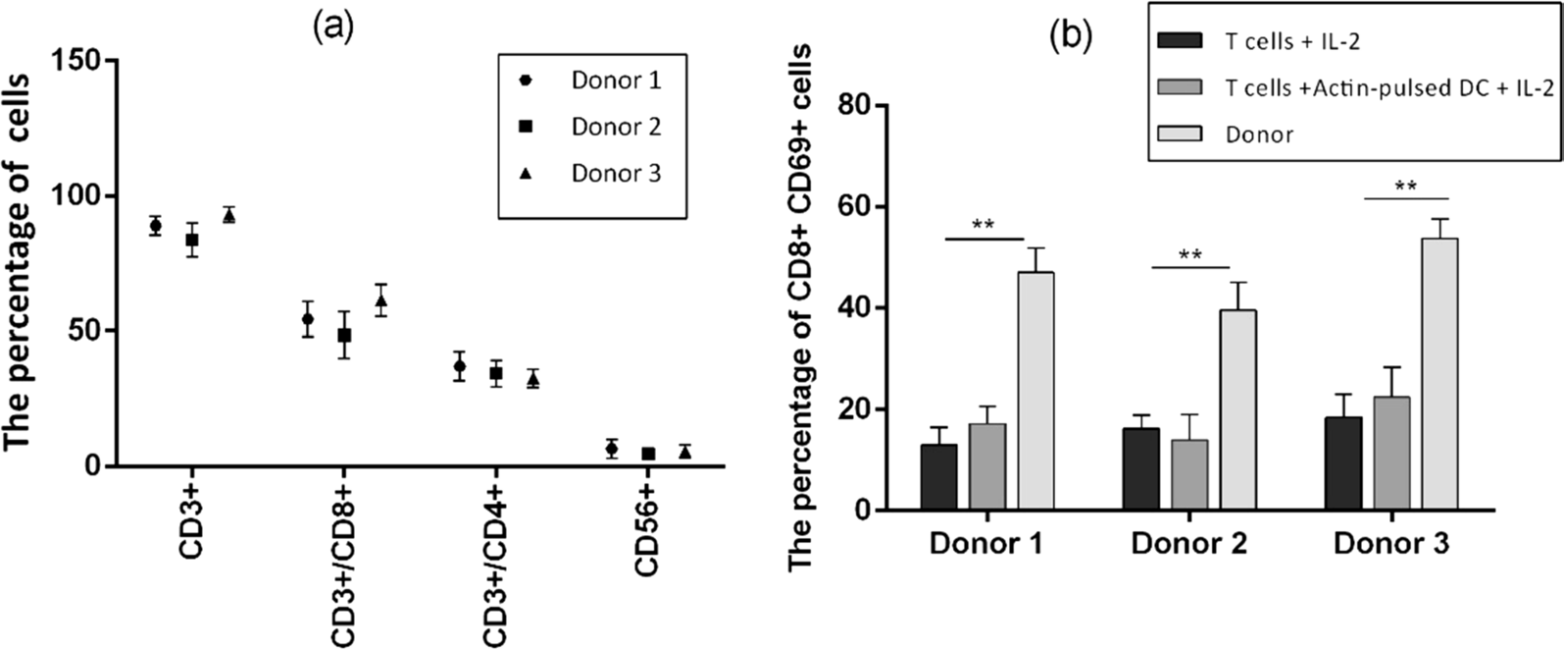
**a** The cell surface marker phenotype of BK-VSTs (*n* = 3) showed both CD4 + T cells and CD8 + T cells and low levels of natural killer (NK) cells (CD56 +). **b** The percentage of CD8 + CD69 + cells on day 14 in donors compared to both controls which is statistically significant (*p* value < 0.01). T cells treated with IL-2 and T cells co-cultured with Actin-pulsed mo-DCs are used as controls

Final VST products were mainly comprised of CD3 + T cells (mean, 88.67% ± 4.76%) (Fig. 5a), with both CD4 + (mean, 34.68% ± 4.46%) and CD8 + (mean, 54.48% ± 8.23%) populations. Natural killer cells were detectable at low levels (mean, 5.75% of lymphocytes ± 2.36%).

We also calculated the percentage of cells that were positive for both CD8 and CD69 (Fig. 5b). The median of 47.0 ± 4.9%, 39.6 ± 5.4%, and 53.7 ± 3.8% of the final cells were positive for both CD8 and CD69 markers on day 14 for donor 1, 2 and 3, respectively, which showed a significant increase compared to both controls (*p* value < 0.01). No significant difference was observed in the percentage of CD8/CD69 double positive cells between the donors. The percentage of CD8/CD69 double positive T cells treated with IL-2 was 15.93 ± 2.7% and 17.9 ± 4.29% for T cells co-cultured with Actin-pulsed mo-DCs.

## Discussion

BKPyV-HC is considered as one of the main HSCT-associated complications with a prevalence rate of about 40% in some cases [10]. It causes remarkable mortality and morbidity. There is still no standard and approved treatment practice for BKPyV-HC, and it is managed based on the local standard operating procedures [6, 12]. Recently, ACT has shown great promise in the fight against transplant-associated viruses [21]. The estimated response rates are between 50 and 90%, depending on manufacturing techniques [1]. This makes ACT a potential mode of therapy in HSCT-associated BKPyV infection. We demonstrated that it is possible to generate functional BKPyV-specific T cells in healthy donors using BKPyV PepMixes.

Our results of the CFSE assay showed that donor lymphocytes are able to proliferate in response to BKPyV PepMixes. We used IL-2 in our study that is a potent mitogen for T cells expansion [22]. We observed that the proliferation rate in donors increased significantly compared to both controls. Dave and colleagues generated multivirus-specific T cells that also targeted the BKPyV. They reported a median of 15-fold expansion in T cells after three stimulations with BKPyV PepMixes [23]. Although we did three stimulations in our study, this rate of expansion is much higher than ours, which is 3.75 ± 0.21. Of course, in Dave's study, IL-7 and IL-15 were also used which enhance T cell expansion and second and third stimulations were done with PepMix-pulsed lymphoblastoid cell lines (LCLs) [23]. We performed all three stimulations with the same antigen-presenting cell (APC). In addition, Emily Blyth et al. also produced BKPyV VSTs with a method similar to ours and observed 5.9-fold expansion. They performed two stimulations with the same APC but increased the IL-2 concentration to 50 U/mL from day 14 [24]. In contrast, we used a concentration of 20 U/mL in all steps. Using low IL-2 concentrations decreases the production cost of VSTs and has the added benefit of reducing negative withdrawal effects of IL-2 [25]. In a study that examined the effect of different concentrations of IL-2 on T cell expansion, a concentration of 50 U/mL was reported to be the optimal [26]. The higher fold expansion in Emily's study is probably due to the higher concentration of IL-2.

We also found that generated lymphocytes are able to produce IFN-γ in response to BKPyV PepMixes, and the number of IFN-γ-producing T lymphocytes in donors is significantly higher than controls. Evaluation of VST responses to viral antigens is frequently performed on IFN-γ secreting cells. For instance, multi-VSTs were recently developed for viral respiratory infections, and IFN-γ ELISpot showed the specificity of the product against different viruses [27]. Furthermore, multi-VST derived from umbilical cord blood was manufactured for post-HSCT viruses, which also included BKPyV. Expanded T cells exhibited specificity to both LT and VP1 antigens by IFN-γ ELISPOT assay [23]. Jongming Li et al. also produced BKPy-VSTs using two peptide epitopes, which are supermotive epitopes. They reported that the frequency of VSTs, analyzed by flow cytometric IFN-γ intracellular cytokine assay, was very low [28]. In their study, monocytes were used as APC, but we used DCs, which are more suitable for VST generation. In addition, they used only two immunodominant epitopes of viral antigens, while we used a mixture of several epitopes. However, they successfully exploited a novel monocyte-based solid phase T cell selection system that can usually increase the frequency of VSTs by > tenfold. This T cell selection system is inexpensive and simple and has the ability to produce large quantity of BKPyV-specific T cells [28].

The final cell product in all three donors consisted of a median of 46.8 ± 7.1% CD8 and CD69 positive T cells. As previously reported, T-cell immunity, especially cytotoxic T lymphocyte (CTL), is crucial for protection against BKPyV, and the feasibility of manufacturing BKPyV-specific CTL lines using wild-type BKPyV strain had been demonstrated [29]. Such studies further confirm our results, except that we used viral PepMixes instead of wild type virus, which can reduce the manufacturing time and could be more practical for clinical applications. We also evaluated CD69 expression which is a membrane bound receptor. It can readily be upregulated upon activation in most leukocytes [30]. It can be discovered as early as 2–3 h after stimulation that expresses even quicker than CD25, which is the reason for its widespread use as an early marker of lymphocyte activation. This marker is also involved in T cell priming and proliferation [30]. Expression of activation markers is not dependent on other effector functions such as cytotoxicity or cytokine secretion, which can be limited to the differentiation state of the T cells (e.g. naïve, central or effector memory). Thus, activation markers allow the complete characterization of the total pool of specific T cells against a specific antigen [31]. Nevertheless, CD69 expression may also be found on non-stimulated T cells, and its upregulation is not exclusively dependent on T cell receptor triggering [31]. The analysis of CD69 expression alone possibly overestimates the frequency of BKPyV-specific T cells. Due to the characteristics of this marker, it is possible to intensify the purity of the antigen-specific population in the final product for clinical applications by using selection techniques such as magnetic-activated cell sorting (MACS).

The immune properties of VSTs are tightly related to their cytotoxicity. We observed that VSTs are able to eliminate BKPyV antigen-expressing target cells. However, we did not measure the survival life time of the VST and the cell viability after the induction. The main purpose of our study was to investigate the feasibility of manufacturing this cell product.

## Conclusion

Considering the fact that several clinical trials have shown the safety and efficacy of using BKPyV VSTs, devising an efficient ex vivo expansion protocol for such T cells is crucial in propelling the widespread use of BKPyV cell therapy. In this study, we demonstrated that it is possible to produce functional BKPyV-specific T cells in healthy donors using BKPyV PepMixes.

## Data Availability

You can email the Corresponding author for more details on the method and data of this paper. The datasets used and/or analysed during the current study available from the corresponding author on reasonable request.
